# DEMENTIA-PROFICIENT MANAGED CARE: ACADEMIC–INDUSTRY COLLABORATION ON A MULTITIERED STAFF TRAINING PROGRAM

**DOI:** 10.1093/geroni/igae098.1815

**Published:** 2024-12-31

**Authors:** David Engle, GeVonne O’Neil, Halima Amjad, Cynthia Fields, Quincy Samus

**Affiliations:** Centene Corporation, House Springs, Missouri, United States; Centene Corporation, St. Louis, Missouri, United States; Johns Hopkins University School of Medicine, Baltimore, Maryland, United States; Johns Hopkins University School of Medicine, Baltimore, Maryland, United States; Johns Hopkins University, Baltimore, Maryland, United States

## Abstract

Over 6.5 million people are living with Alzheimer’s Disease and related dementias (ADRD) in the U.S. Effective care management can improve care quality, quality of life, delay institutionalization, and decrease unnecessary care costs. Centene Corporation, a leading US health care enterprise, operates government-sponsored healthcare programs and offers coverage to 27.5 million managed care members, including Medicaid and Medicare members. Centene and Johns Hopkins University have been engaged in an academic-industry collaboration since 2019 to address the increase in Centene’s membership population who are living with ADRD, promote dementia care proficiency within care teams, and drive person-centered care to help members achieve their care goals and exercise control over their services. This presentation will describe the development process, content, and nation-wide deployment of a new, evidence-based multi-tiered dementia care specialist (DCS) training program for managed care and health plan teams. The DCS covers 7 learning areas and aligns with CMS and state training requirements. We will review eLearning and live instructor-led modules, how the train-the-trainer program was implemented in practice settings engaging over 500 learners across 37 states since October 2022, and summarize assessment results on key metrics showing a nearly 50% increase proficiency on measures, before to after training.

